# Incorporating the field border effect to reduce the predicted uncertainty of pollen dispersal model in Asia

**DOI:** 10.1038/s41598-021-01583-x

**Published:** 2021-11-12

**Authors:** Yuan-Chih Su, Cheng-Bin Lee, Tien-Joung Yiu, Bo-Jein Kuo

**Affiliations:** 1 Department of Agronomy, National Chung Hsing University, Taichung, 402202 Taiwan, ROC; 2 Division of Biotechnology, Taiwan Agricultural Research Institute, Taichung, 41362 Taiwan, ROC; 3 Tainan District Agricultural Research and Extension Station, COA, Executive Yuan, Chiayi, 613014 Taiwan, ROC; 4 Innovation and Development Center of Sustainable Agriculture (IDCSA), National Chung Hsing University, Taichung, 402202 Taiwan, ROC; 5 Pervasive AI Research (PAIR) Labs, Hsinchu, 30010 Taiwan, ROC

**Keywords:** Biotechnology, Ecology, Plant sciences

## Abstract

The presence of the field border (FB), such as roadways or unplanted areas, between two fields is common in Asian farming system. This study evaluated the effect of the FB on the cross-pollination (CP) and predicted the CP rate in the field considering and not considering FB. Three experiments including 0, 6.75, and 7.5 m width of the FB respectively were conducted to investigate the effect of distance and the FB on the CP rate. The dispersal models combined kernel and observation model by calculating the parameter of observation model from the output of kernel. These models were employed to predict the CP rate at different distances. The Bayesian method was used to estimate parameters and provided a good prediction with uncertainty. The highest average CP rates in the field with and without FB were 74.29% and 36.12%, respectively. It was found that two dispersal models with the FB effect displayed a higher ability to predict average CP rates. The correlation coefficients between actual CP rates and CP rates predicted by the dispersal model combined zero-inflated Poisson observation model with compound exponential kernel and modified Cauchy kernel were 0.834 and 0.833, respectively. Furthermore, the predictive uncertainty was reducing using the dispersal models with the FB effect.

## Introduction

Maize (*Zea mays* L.) is one of the most important genetically modified (GM) crops globally, and the area of GM maize accounts for 32% of the total GM crop area^1^. Although GM crops are crucial in the global crop production, the safety of GM products is still debated between producers and consumers, even though GM products have been on the market for two decades^2^. The labeling threshold was established to protect non-GM crops and products admixed by the adventitious presence (AP) of GM content^3^.

The European Food Safety Authority indicated that the risks of GM plants in regard to persistence and invasiveness have to be assessed^4^. The outcrossing with wild or weedy relatives is a way to transfer the GM content to the environment^5,6^. The hybridization of GM crops and wild relatives has been studied in the past^7,8^. The study has indicated that the GM maize will hybridize with landrace^9^. The escape of GM content to the environment may reduce of diversity of flora and make weed control harder^4^. Therefore, the assessment of environment risk of GM crops should be concerned.

When conventional crop production cannot yield food sufficiently for the entire population, producers and consumers must seek solutions from other crop production systems. GM crops provide a solution for feeding the growing population^10^. Therefore, the coexistence between GM and non-GM crop production systems has been studied in past 2 decades. Coexistence denotes that farmers can choose between organic, non-GM, and GM crop production systems, but the GM contents of the products must meet the labeling standard^11^. A practical strategy of coexistence would minimize the economic loss and cost.

Pollen dispersal is the main source of AP in the maize production. Not only the pollen dispersal of maize but also the other species, such as tree, had been studied^12^. There are many factors (e.g. meteorology, topography) that can affect the pollen dispersal^13–15^. The emission of pollen is affected by temperature and humidity^16^. The pollen emission period is longer when the temperature is low. The onset of pollen emission will be delayed when the humidity is high. The size of pollen influenced the pollen setting velocity, but the genetic variation among pollens from the same plant and different plants was found^17^. The maize has a relative high setting velocity because of the pollen size. In general, the result of pollen dispersal varies widely with the environments and the plants.

Setting isolation distances to avoid the AP caused by the pollen dispersal is a common containment strategy in numerous countries. However, a fixed isolation distance is not suitable for different cultivation scenarios and may reduce the profit of production. In Asia, most fields are small and close to each other. The fields are usually separated by the field border (FB), such as roadways between two fields or unplanted areas. Under this kind of cropping system, farmers must pay a higher cost of coexistence than extensive farming systems. Studies have indicated that the main factors affecting the AP in a small field were the synchronicity of flowering and isolation distance^18^. The flowering time isolation is an alternative strategy to cope with the limitation of the field size^19^. However, the separation of flowering time may cause an unfavorable growing season^20^. Accurate prediction of the AP can provide the useful information for the establishment of the isolation distances. Numerous pollen dispersal models including exponential, log/square, log/log, Gaussian plume, and two-step model were developed to predict the AP caused by the pollen dispersal^21–23^.

The present study conducted three experiments to simulate different Asian cultivation scenarios. Different dispersal models were established to predict the CP rate. Specifically, the FB effect was included in the dispersal models to adapt to the Asian farming system. Additionally, the predicted uncertainty was evaluated using the Bayesian estimation method.

## Methods

### Dispersal models

**Figure 1:**
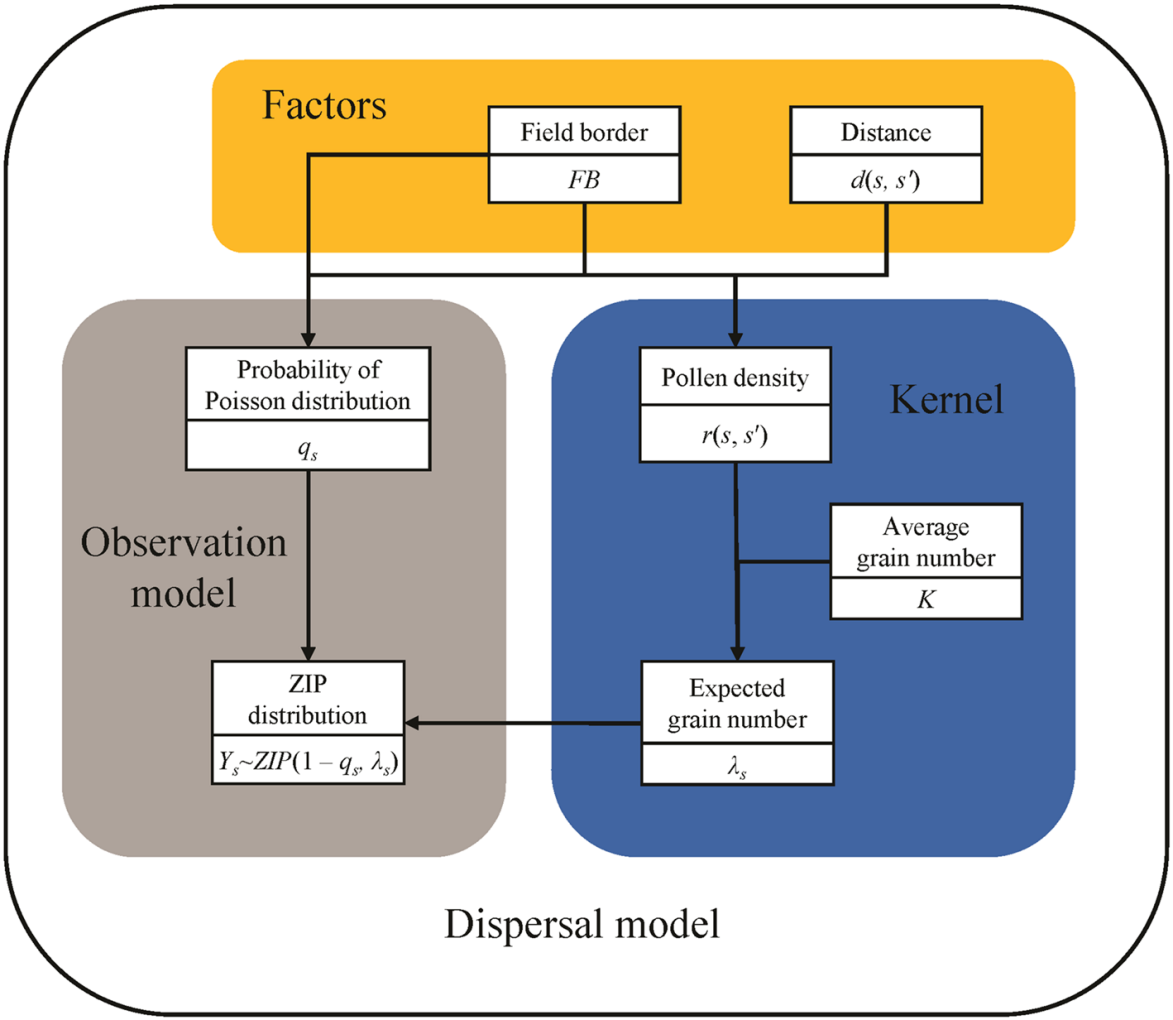
Graphical summary of the establishment of the dispersal model using ZIP distribution observation model as an example.

In this study, the dispersal model consists of two parts, namely, kernel and observation model (Fig. 1). The main purpose of the kernel was employed to estimate the proportion of pollen dispersed from location *s*′ to location *s* and calculate the expected number of CP grains. The observation model used the expected number of CP grains as a parameter and described the number of CP grains at location *s* (*Y*_*s*_) by a specific distribution in the following:

1 $${Y}_{s}\sim f\left(\left.{y}_{s}\right|{{\varvec{\theta}}}_{s}\right),$$

where *f* indicates the probability density function (PDF) of the specific distribution. The ***θ***_*s*_ is the parameter vector of the distribution. This study constructed eight different dispersal models combined with two observation models, two kernels, and two conditions of the field border (FB) effect (Table 1). The details of the kernels and observation models were described in the following subsections.

**Table 1:** List of dispersal models constructed in this study.

Observation model	Kernel	Field border effect	Model code
Poisson	Compound exponential	−	PExpoN
Poisson	Compound exponential	+	PExpoB
Poisson	Modified Cauchy	−	PCauchyN
Poisson	Modified Cauchy	+	PCauchyB
Zero-inflated poisson	Compound exponential	−	ZExpoN
Zero-inflated poisson	Compound exponential	+	ZExpoB
Zero-inflated poisson	Modified Cauchy	−	ZCauchyN
Zero-inflated poisson	Modified Cauchy	+	ZCauchyB

### Kernels

The kernel indicates the probability when the pollen emitted at location *s*′ and would fall down at location *s*. It can be expressed as γ(*s*, *s*′), where *s*′ is the source location closest to location s. Numerous kernels have been used to describe various dispersal phenomena^24^. The output of the kernel represents the donor pollen density of location *s*. In order to calculate the expected number of CP grains, the donor pollen density is multiplied by the average total grain number described as follows:

2 $${\lambda }_{s}=K\times \gamma \left(s,{s}^{^{\prime}}\right),$$

where λ_s_ and K indicate the expected number of CP grains at location s and the average number of grains per cob, respectively. The effect of the FB was introduced into the kernel to suit to the small-scale farming system in Asia. This study assumed that the relation between the pollen density at the first recipient row and the width of the FB displayed an exponential decrease^25,26^. To evaluate the improvement of the kernel with the FB effect, the kernels without the FB effect were also established in this study.

The compound exponential kernel (γ_Expo_) has been used in the previous pollen dispersal study^27^. Our study introduced the FB effect into this kernel. Therefore, the form of the compound exponential kernel can be expressed as follows:

3 $$\gamma_{{{\text{Expo}}}} \left( {s,s^{\prime}} \right) = \left\{ {\begin{array}{*{20}l} {K_{e} \exp \left( { - a_{1} d^{*} \left( {s,s^{\prime}} \right)} \right)\exp \left( { - k\sqrt {FB} } \right),} \\ {K_{e} \exp \left( { - a_{1} D - a_{2} \left( {d^{*} \left( {s,s^{\prime}} \right) - D} \right)} \right)\exp \left( { - k\sqrt {FB} } \right),} \\ \end{array} } \right.\begin{array}{*{20}l} {{\text{if}}\,\, d^{*} \left( {s,s^{\prime}} \right) \le D} \\ {{\text{if}} \,\,d^{*} \left( {s,s^{\prime}} \right) > D,} \\ \end{array}$$

where *K*_*e*_, *a*_1_, *a*_2_, *k*, *D* are the parameters of the kernel. d*(*s*, *s*′) indicates the shortest distance between locations *s*′ and *s* in which the width of the FB has been subtracted. In the compound exponential kernel without the FB effect, the exponential term of the FB effect was removed and the d*(*s*, *s*′) was replaced directly by the shortest distance between *s*′ and *s*.

The second kernel applied in this study was the modified Cauchy kernel (γ_Cauchy_) which was based on the PDF of the Cauchy distribution and the concept of compound distribution. The modified Cauchy kernel is represented as follows:

4 $$\gamma_{Cauchy} \left( {s,s^{\prime}} \right) = \left\{ {\begin{array}{*{20}l} {\frac{2\beta }{{\pi \left[ {\beta^{2} + d^{*} \left( {s,s^{\prime}} \right)^{2} } \right]}}{\text{exp}}\left( { - k\sqrt {FB} } \right),} \\ {\frac{2\beta }{{\pi \left[ {\beta^{2} + D^{2} + c_{1} \left( {d^{*} \left( {s,s^{\prime}} \right) - D} \right)^{2} } \right]}}{\text{exp}}\left( { - k\sqrt {FB} } \right),} \\ \end{array} } \right.\begin{array}{*{20}l} {{\text{if}} \,\,d^{*} \left( {s,s^{\prime}} \right) \le D} \\ {{\text{if}} \,\,d^{*} \left( {s,s^{\prime}} \right) > D,} \\ \end{array}$$

where the *β* indicates the decline rate of the curve. Parameters of *k* and *D* are same as the compound exponential kernel. *c*_1_ indicates the relative slow decrease of pollen density at further distances. Similarly, in the modified Cauchy kernel without the FB effect, the term of the FB effect was removed and the d*(*s*, *s*′) was replaced directly by the shortest distance between *s*′ and *s* in which the row spacing (0.75 m) had been subtracted.

### Observation models

Because of the high proportions of zero value observations, the present study assumed that the CP grain count followed the zero-inflated Poisson (ZIP) distribution to account for zero-excess condition^28^. The ZIP distribution was first proposed by Lambert^29^, and several studies had applied the ZIP distribution to deal with the CP data^27,30^. The ZIP distribution consists of a Dirac distribution in zero and a Poisson distribution. Therefore, the distribution of CP grain count at location *s* (*Y*_*s*_) can be expressed as follows:

5 $${Y}_{s}\sim \mathrm{ZIP}\left(1-{q}_{s},{\uplambda }_{s}\right),$$

where *q*_*s*_ indicates the probability of an observation following a Poisson distribution, and *λ*_*s*_ is the parameter of Poisson distribution calculated by Eq. (2). Furthermore, the parameter *q*_*s*_ can be assumed to depend on the shortest distance between the recipient and donor plants. The border effect is also included in the estimation of *q*_*s*_ because it is related to the distance effect. The relationship among distance, border, and the *q*_*s*_ can be described using the following logistic function:

6 $${q}_{s}=\frac{1}{1+\mathrm{exp}({b}_{1}-{b}_{2}{d}^{*}\left(s,{s}^{^{\prime}}\right))},$$

where *b*_1_ and *b*_2_ are the parameters of the logistic function. The d*(*s*, *s*′) was the shortest distance between *s*′ and *s* in the version of dispersal models without the FB effect. The Poisson distribution was also used as an observation model for comparison with the ZIP observation model.

### Experimental and meteorological data collection

The pollen dispersal data were collected from experiments performed in 2009 and 2010 at the geographic coordinates 23° 47′ N, 120° 26′ E, and an altitude of 20 m. These experiments were coded as 2009-1, 2009-2, and 2010-1, respectively. The experiment 2009-2 was divided into 2009-2A (without the FB) and 2009-2B (with the FB) based on the presence of the FB. The different layouts of the field experiments were designed to investigate the effect of the FB. Two commercial glutinous maize varieties, black pearl (purple grain) and Tainan No. 23 (white grain), were selected as the pollen donor and pollen recipient, respectively. The distance between the plants in a row was 25 cm, whereas the distance between the rows was 75 cm. The recipient plots consisted of 82 and 91 rows in 2009 and 2010 experiments, respectively.

The CP rate was determined based on the differences in grain color on recipient cobs as a result of the xenia effect^31^. In the sampling framework, the whole field was divided into many grids and corn samples were collected from each grid in the whole field. The CP rate of each grid was calculated using the method presented in a previous study^32^ and defined as:

7 $$\mathrm{CP}\left(\%\right)=\left[\sum_{i=1}^{n}{Cob}_{i}/\left(n\times K\right)\right],$$

where *Cob*_*i*_ and *n* indicate *i*th cob and total number of cobs in the grid, respectively. *K* is the average grain number per cob. Meteorological data were collected from the meteorological station at geographic coordinates 23° 35′ N, 120° 27′ E, and an altitude of 20 m. The detailed experimental setup was described in our previous study^33^. The study complies with relevant institutional, national, and international guidelines and legislation.

### Statistical analyses

All statistical analyses were performed using SAS (Statistical Analysis System, version 9.4). The dispersal model parameters were estimated by two methods. First, the nonlinear model estimation was conducted by PROC NLMIXED to evaluate the fitting and predictive abilities of dispersal models. Then the dispersal models with the observation model performed better fitting ability were re-estimated using the Bayesian estimation method to assess the uncertainty by PROC MCMC. In the Bayesian method, the noninformative prior distribution was used to estimate all parameters (Supplementary Table S1). The iteration of Markov Chain was 500,000 times and the burn-in was set to 450,000 iterations. In order to reduce the autocorrelations in the chain, the thinned value was set to 25.

The validation method used in this study was the threefold cross-validation for the results of both estimation methods. The data from three experiments were combined and randomly partitioned into three sub-datasets. To avoid the heterogeneity of the different field designs and distances among sub-datasets, the observations from the same field design and same distance were considered as a group, and then partitioned into three parts. Each sub-dataset contained one part of all groups. At each validation run, two sub-datasets were selected as the training set, and the remaining one was used for validation.

The fitting ability of the dispersal models was evaluated based on two criteria, namely, Akaike information criterion (AIC), Deviance, and coefficient of determination (R^2^). The smaller values of AIC or deviance indicate a better fitting. The higher R^2^ value represents a better fitting performance. The correlation coefficient (r) between the predicted and actual CP rates was used to assess the predictive ability. The deviance information criterion (DIC) was used to evaluate the performance of dispersal model fitting for the Bayesian estimation. The criterion values calculated from three training and validation sets were averaged to assess the overall results. The uncertainty of the model parameter was quantified by the standard deviation (SD) of parameter posterior distribution. The 95% credible intervals of posterior predictive distribution constructed by the 2.5th and 97.5th percentiles of 200,000 samples generated from the posterior predictive distribution were used to assess the predictive uncertainty. Furthermore, to assess the zero-excess condition, the percentage of observed zero CP grain events was compared with the Poisson probability of the zero CP grain event. A zero-excess condition occurred if the observed percentage was higher than the Poisson probability^34^.

## Results

### Cross-pollination

**Figure 2:**
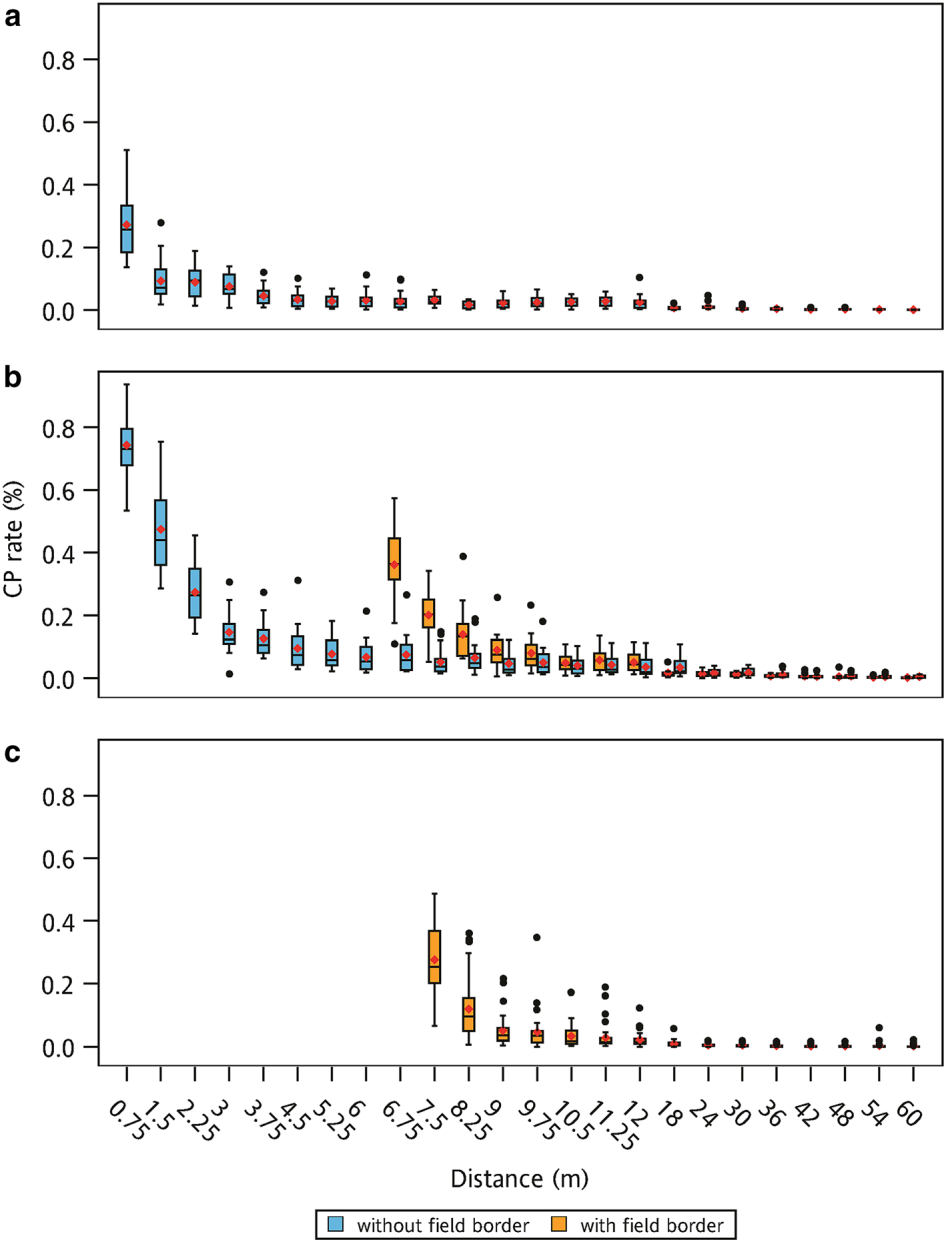
Box plots of cross-pollination (CP) rate of (**a**) 2009-1 experiment, (**b**) 2009-2 experiment, and (**c**) 2010-1 experiment. The red diamonds and black dots represent the mean and outliers, respectively.

The highest CP rate of each experiment was observed in the first row of the recipient plot (Fig. 2). Compared with the average CP rate at the first row, the average CP rate declined by 50% in the second or third recipient row. In the 2009-1 experiment, the average CP rates at 0.75 and 1.5 m were 27.24% and 9.35%, respectively. The average CP rates of the first row in the 2009-2A and 2009-2B experiments were 74.29% (0.75 m) and 36.12% (6.75 m), respectively. The average CP rates decreased by 50% in the third row in the 2009-2A and 2009-2B experiments. The average CP rate in the first row (7.5 m) was 27.58% and dropped to 11.97% in the second row (8.25 m) in the 2010-1 experiment. In three experiments, the CP rate drastically decreased in the first few rows. However, the CP rate still could be observed at the farthest sampling points.

### Field border effect and zero-excess

In the 2009-2A and 2009-2B experiments, the difference in the average CP rate at a distance of 6.75 m indicated that the CP rate was different between the field with and without FB (Fig. 1). The average CP rates of experiments 2009-2A and 2009-2B at 6.75 m were 7.43% and 36.12%, respectively. The field without FB had a larger reduction in the CP rate compared with the field with FB. The difference in the CP rate between experiments 2009-2A and 2009-2B was observed within 30 m.

In this study, the zero-excess situations at different distances were also examined. We evaluated the level of the zero-excess of each experiment by the proportion of zero-excess condition of whole field. The probability of zero CP of each distance was calculated and compared with the proportion of zero CP event of actual data. The zero-excess levels differed among three experiments (Supplementary Table S2). The zero CP grain event mainly occurred at the distance > 10 m. The 2010-1 experiment exhibited the highest zero-excess level, and the percentage of the zero-excess condition was 60%. However, the zero-excess levels in other experiments were relatively low (2009-1: 20.8%; 2009-2A: 12.5%; 2009-2B: 6.25%).

### Fitting and predictive abilities of dispersal models

This study constructed eight dispersal models including different observation models, kernels, and with and without the FB effect. According to the AIC, deviance, and R^2^, the dispersal models with the FB effect had a better fitting ability than the dispersal models without the FB effect (Table 2). The fitting ability of modified Cauchy kernel without the FB effect was superior to that of the compound exponential kernel without the FB effect. However, the compound exponential kernel with the FB effect displayed a better fitting ability than the modified Cauchy kernel with the FB effect. The dispersal models with ZIP observation model performed a better fitting ability than the dispersal models with Poisson observation model. Overall, the ZExpoB model showed the best fitting ability and the ZCauchyB was the second. Additionally, the results of the predictive ability and fitting ability among the dispersal models were similar. However, the difference of the predictive ability of dispersal models with Poisson and ZIP observation models was negligible. The PCauchyB and ZExpoB models performed the best predictive ability and both models had a same correlation coefficient of 0.834.

**Table 2:** The average mean and standard deviation of Akaike information criterion (AIC), deviance, coefficient of determination (R^2^), and correlation coefficient (r) of dispersal models obtained using threefold cross-validation.

Model code	AIC	Deviance	R^2^	r
PExpoN	47,162 ± 669	35,014 ± 463	0.579 ± 0.025	0.76 ± 0.036
PExpoB	36,647 ± 784	24,497 ± 504	0.703 ± 0.006	0.833 ± 0.013
PCauchyN	44,227 ± 861	32,081 ± 737	0.582 ± 0.028	0.762 ± 0.033
PCauchyB	37,475 ± 1113	25,327 ± 833	0.696 ± 0.007	0.834 ± 0.009
ZExpoN	45,856 ± 639	33,207 ± 432	0.574 ± 0.025	0.76 ± 0.036
ZExpoB	35,780 ± 784	22,899 ± 548	0.701 ± 0.007	0.834 ± 0.012
ZCauchyN	43,608 ± 994	31,141 ± 1065	0.579 ± 0.022	0.763 ± 0.031
ZCauchyB	36,588 ± 1053	23,922 ± 880	0.691 ± 0.011	0.833 ± 0.01

**Figure 3:**
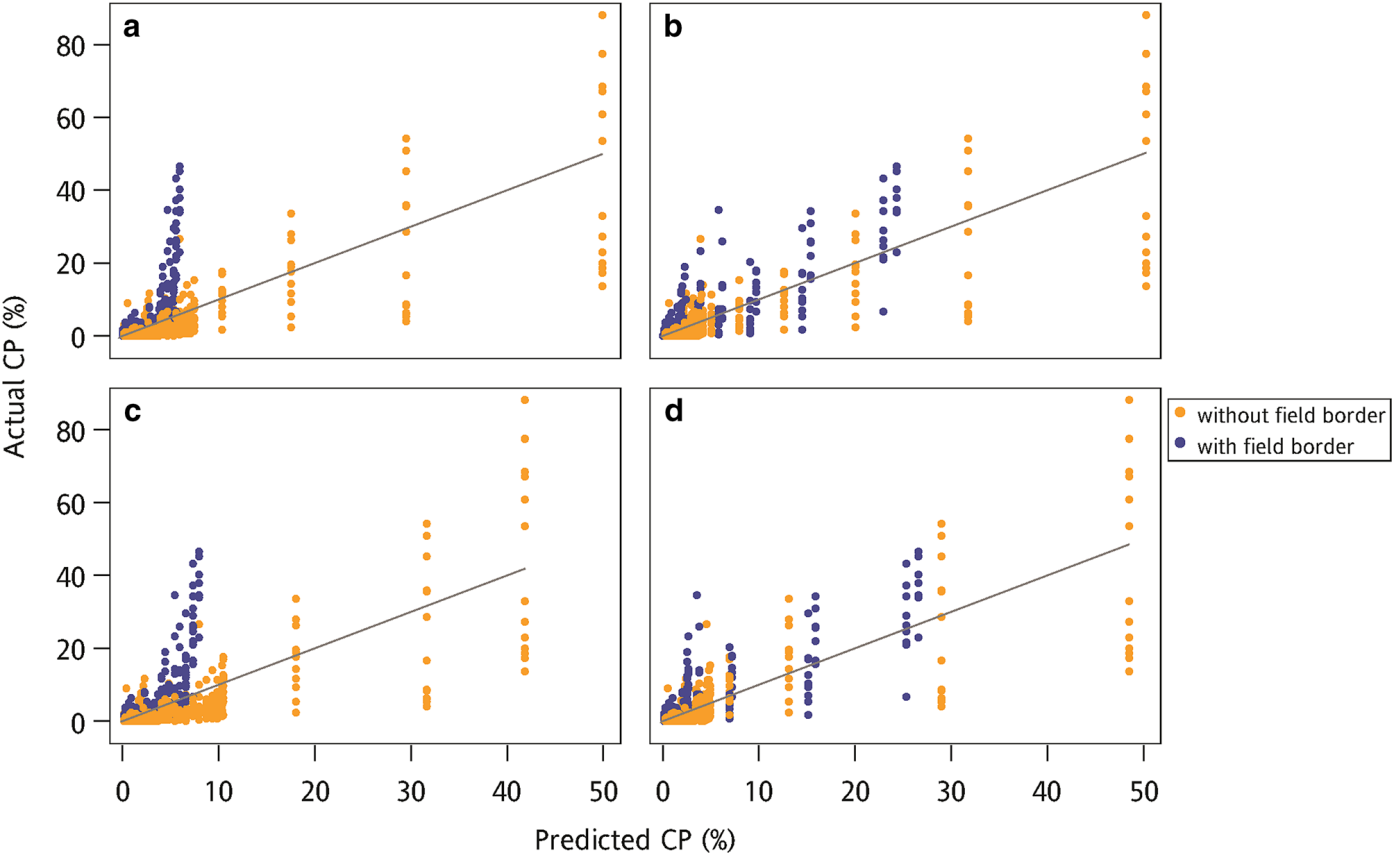
Scatter plots of the actual cross-pollination (CP) rate from the validation data and predicted CP rate using the (**a**) ZExpoN, (**b**) ZExpoB, (**c**) ZCauchyN, and (**d**) ZCauchyB models to fit the training data. The gray line is the 1:1 reference line.

According to the fitting result of dispersal models, the dispersal models with the ZIP observation model were the preferred models for describing the CP data. Therefore, the dispersal models with the ZIP observation model were selected to investigate the difference of predicted CP rate between the dispersal models with and without the FB effect. The scatter plot of the actual CP rate from the validation data versus the predicted CP rate calculated using four dispersal models fitted with the training data was represented in Fig. 3. It was found that the dispersal model without the FB effect seriously underestimated some CP rates from the field with FB (Fig. 3). In contrast, the predicted CP rate from the dispersal model with the FB effect distributed around the reference line evenly. The result indicated that the dispersal models with the FB effect had a great predictive ability under the conditions of the field with and without FB.

### Predicted uncertainty of dispersal models

The parameters of dispersal models with the ZIP observation model were re-estimated by the Bayesian estimation method. According to the DIC, the ZExpo model appeared the best fitting ability, with the ZCauchy model following (Supplementary Table S3). The comparison result of fitting ability between dispersal models estimated by Bayesian method was similar to the result by nonlinear estimation.

**Figure 4:**
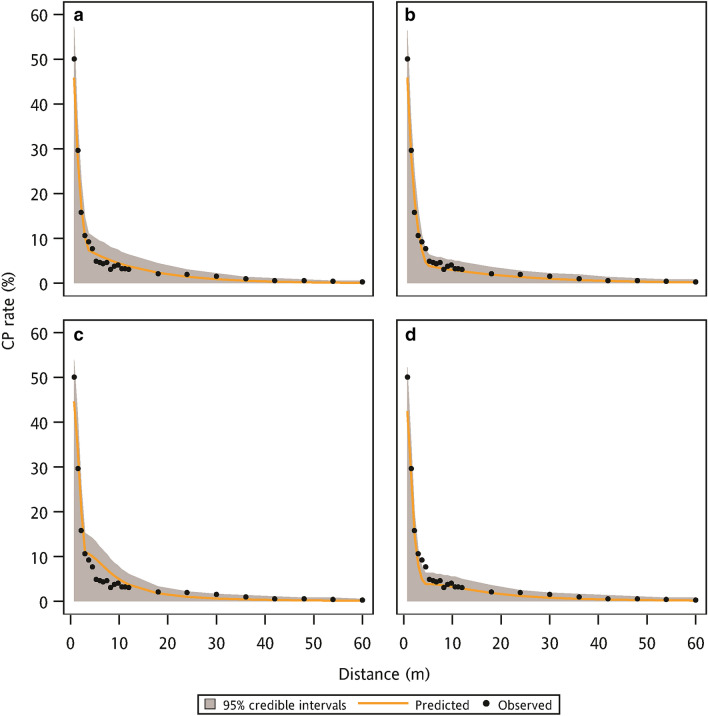
The observed CP rate from validation data (without FB condition) and the predicted CP rate with 95% credible intervals using the (**a**) ZExpoN, (**b**) ZExpoB, (**c**) ZCauchyN, and (**d**) ZCauchyB models to fit the training data.

**Figure 5:**
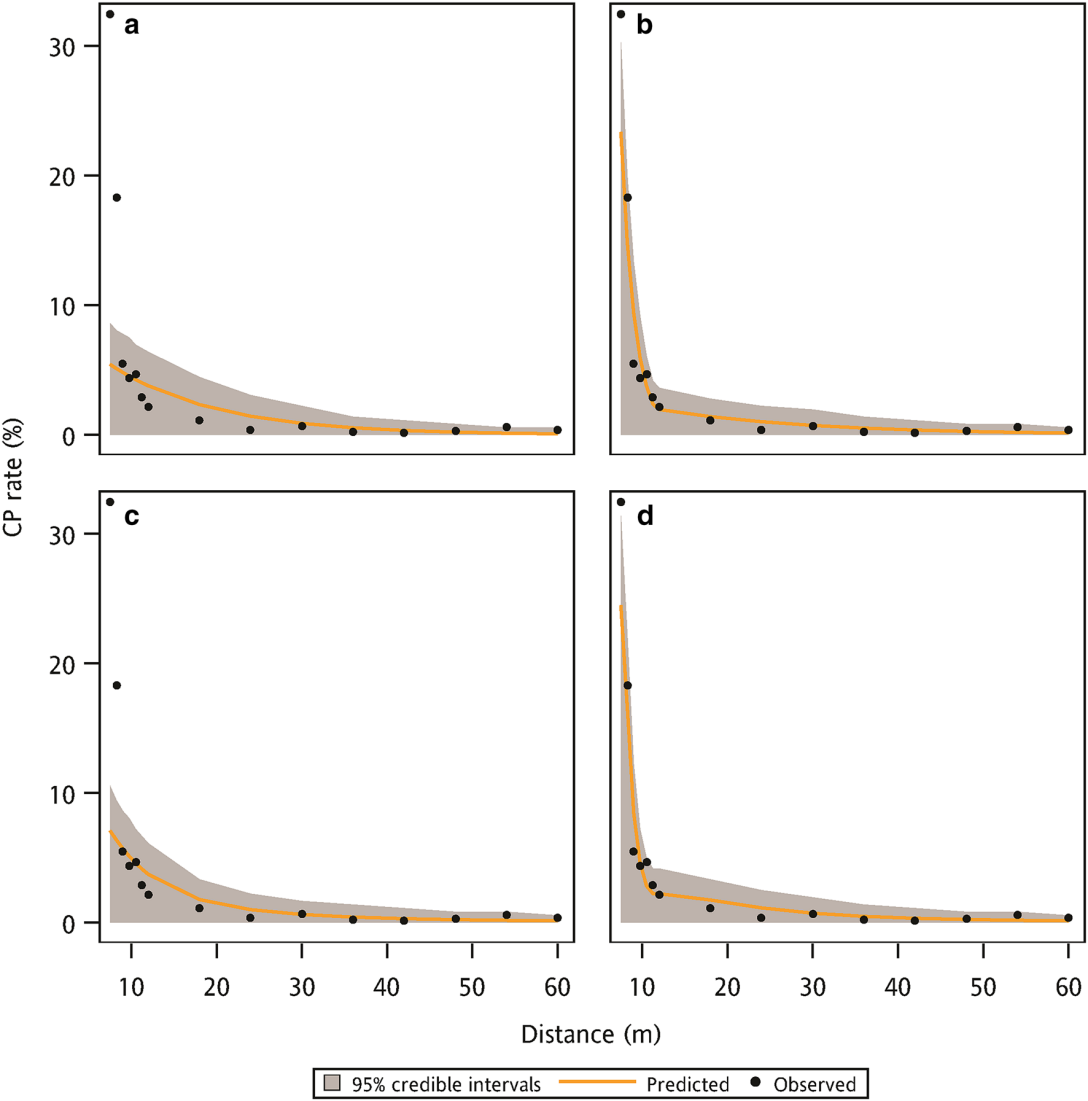
The observed CP rate from validation data (with 7.5 m FB condition) and the predicted CP rate with 95% credible intervals using the (**a**) ZExpoN, (**b**) ZExpoB, (**c**) ZCauchyN, and (**d**) ZCauchyB models to fit the training data.

For the parameter estimation of the Bayesian method, the average mean values of the parameter posterior distribution between ZExpoN and ZExpoB models were comparable (Table 3). However, the ZExpoB model had a small SD of the parameter posterior distribution than the ZExpoN model. This result indicated the posterior distribution of parameters between these two models were similar but the parameter uncertainty of ZExpoB model was smaller than that of ZExpoN model. The ZCauchyB model also performed a small parameter uncertainty, but only a half of the SD of the parameter posterior distribution was reduced after adding the FB effect (Table 4). The predicted uncertainty was assessed under the field conditions with and without FB and quantified by the 95% credible interval of posterior predictive distribution of the CP rate. The posterior predictive distribution of the CP rate was generated by the dispersal models fitted with the training data. Under the field condition without FB, dispersal models with the FB effect illustrated a relatively narrow 95% credible interval, especially at the distance between 3 and 10 m (Fig. 4). The narrow 95% credible intervals indicated a smaller predicted uncertainty. The predicted CP rates calculated by four dispersal models were closed to the observed CP rates of the data points without FB from the validation data. For the field condition with a 7.5 m width FB, ZExpoB and ZCauchyB models performed a smaller predicted uncertainty at the distance between 7.5 m and 18 m than ZExpoN and ZCauchyN model (Fig. 5). The predicted CP rates were compared to the observed CP rates from the validation data that only included the data points with the 7.5 m FB. In addition, the dispersal models without the FB effect underestimated the CP rates at distances of 7.5 m and 8.25 m. These results showed that the dispersal models with the FB effect could reduce the predicted uncertainty at the field conditions with and without FB.

**Table 3:** The average mean and standard deviation (SD) of parameter posterior distribution of ZExpoN and ZExpoB models obtained using threefold cross-validation.

Parameter	Model code			
	ZExpoN		ZExpoB	
	Mean	SD	Mean	SD
K_e_	0.8318	0.0174	0.6760	0.0091
a_1_	0.6823	0.0131	0.6073	0.0065
a_2_	0.0781	0.0042	0.0506	0.0007
k	n/a	n/a	0.3552	0.0060
D	3.3954	0.0488	2.8480	0.0437
b_1_	2.5743	0.1226	2.7621	0.1103
b_2_	0.0086	0.0039	0.0275	0.0034

**Table 4:** The average mean and standard deviation (SD) of parameter posterior distribution of ZCauchyN and ZCauchyB models obtained using threefold cross-validation.

Parameter	Model code			
	ZCauchyN		ZCauchyB	
	Mean	SD	Mean	SD
β	1.3608	0.0196	1.1000	0.0122
c_1_	0.1467	0.0028	0.2832	0.0038
k	n/a	n/a	0.0632	0.0016
D	2.4030	0.0257	3.5803	0.0401
b_1_	3.0979	0.1448	2.7795	0.1102
b_2_	0.0927	0.0044	0.0266	0.0034

## Discussion

This study conducted three experiments with different field designs to investigate the effect of FB on CP results. The main difference between the field designs was with or without the setting of the FB. A study by Della Porta et al.^21^ indicated that maize plants were a more efficient barrier against the CP than the unplanted area. Because the pollen was obstructed by maize plants, the CP rate at the first row in the plot with the FB was lower than the CP rate at the first row in the plot without the FB. Under the 0.9% threshold level, fields with the unplanted area (25.9 m) required a longer isolation distance than fields with the planted area (17.5 m), because the intervening plants between source and recipient fields compete with their own pollens. This difference between the planted and unplanted areas was also observed in a study on oilseed rape^35^. Our findings also revealed a similar declining trend in CP rates in fields with and without FB. Furthermore, the result of the oilseed rape study also supported the hypothesis that the border and buffer zone should be treated as different factors in small-scale field condition studies^35^. Moreover, they found if the dispersal model without the FB effect, the CP rate was underestimated. This is consistent with the result of our study. The CP results of the pollen dispersal experiment and the predictive ability of the fitted model usually depend on the arrangement of the source and recipient fields^23,36^. The self-protection index proposed by Melé et al.^37^ demonstrated that recipient fields with a large area and a small perimeter displayed a higher CP protection. Because of the self-protection ability, the CP results in a large-scale experiment are not affected by the presence of the FB effect. However, the CP results of our study showed a notable FB effect. Therefore, the fields with and without the FB effect would result in different CP trends in the small-scale experiment.

When the number of zero in the data is more than the excepted number of zero in Poisson distribution, the problem of zero-excess occurs. The zero-excess characteristic of the dispersal data has been investigated in some studies^38,39^. Because the recipient plots were set downwind of the source plots, the excess level of the zero value differed in the experiments in our study. This arrangement of the recipient plot can explain the low zero-excess level in some of our experiments. The obvious fluctuation of the level of zero-excess among experiments probably due to the high variation of CP grain number at the long distance. According to the result of Bensadoun et al.^27^, the CP grain number variation was higher at a long distance in the downwind direction plots. The higher level of zero-excess of experiment 2010-1 might result from the sampling bias.

The dispersal models were constructed by combining different observation models and kernels in this study. The zero-inflated models, such as the ZIP mode and zero-inflated negative binomial model, were usually used to describe the zero-excess count data^27,30^. Bensadoun et al.^27^ reported that the cross validation result of the dispersal model with the ZIP observation model (R^2^ = 0.582) was better than that of the Poisson observation model (R^2^ = 0.511). However, in our study, the dispersal models with the Poisson and ZIP observation models had a negligible difference in the R^2^ values. For the models of PExpoN and ZExpoN, R^2^ values were close to the 0.582. It was concluded that this negligible difference resulted from the low zero-excess level in some experimental data.

Numerous fat-tailed kernels have been used in dispersal studies, such as Cauchy^40^, log-sech^30^, and 2Dt^41^. Paradis et al.^42^ reported that the Cauchy kernel predicted long-distance dispersal events more frequently than the exponential model. Studies have used only the scale parameter in the Cauchy kernel to describe the dispersal result^42,43^. The present study modified the Cauchy kernel and retained the location parameter of Cauchy kernel to introduce the FB effect. The FB effect enabled the modified Cauchy kernel to be more suitable to fit the data for the field designs with and without FB. In this study, the FB effect was also introduced into the compound exponential kernel to compare with the modified Cauchy kernel. When incorporating the FB effect into the dispersal models, the predictive ability was increased. The dispersal models with the modified Cauchy kernel (r = 0.833) and the compound exponential kernel (r = 0.834) displayed favorable results in the CP rate prediction. As described in our previous study, adding the FB effect into the models also improved the predictive ability of models^33^. In another study, the highest R^2^ of the fitting result of log/log model was 0.91, when the field included a 17 m empty space between the source and recipient^21^. For a farm scale study, R^2^ of the two-step model was 0.997^23^. Although aforementioned models had a great fitting ability, the predictive ability should be further validated by a suitable validation procedure. More importantly, those models might fail to predict the CP rate because of only considering the distance effect.

The uncertainty was rarely mentioned in previous pollen dispersal studies. The study indicated that when the wind effect was considered in the dispersal model, the predicted uncertainty was decreased^44^. Similarly, the FB effect was incorporated into the dispersal models to reduce the predicted uncertainty in our study. Therefore, this finding indicates that adding some effects which could improve the ability of dispersal model to adapt to various scenarios also might diminish the predicted uncertainty.

## Conclusion

The setting of isolation distance has relative high cost in small-scale farming system despite that the isolation distance is the main strategy of the coexistence. Small-scale farming systems with the FB and the planted area display notable differences in CP rates. In fact, FBs can be regarded as a short isolation distance. Therefore, the combination of plant date and FB may be a measure instead of the isolation distance in small-scale farming system. Because of the difference of CP rates between the FB and planted area, predictions of CP rates should account for the FB effect and make a distinction between the FB and planted area in small-scale fields. Incorporating FB effect into the dispersal models improved both the fitting ability and the predictive ability. In addition, the dispersal models with FB effect predicted the CP rates with a relatively small uncertainty. The introducing of Bayesian estimation can not only evaluate the predictive uncertainty, but also provide a prediction with a probabilistic support.

## Supplementary Information

Supplementary Tables.

## Data Availability

The data presented in this study are openly available in FigShare at https://doi.org/10.6084/m9.figshare.13370756.v1.
