# Supplementary material for: Incorporating the field border effect to reduce the predicted uncertainty of pollen dispersal model in Asia
Source: Sci Rep. 2021 Nov 12;11:22187. doi: 10.1038/s41598-021-01583-x (PMC8589847; doi:10.1038/s41598-021-01583-x)
Supplement: Supplementary file 1 — Supplementary Tables. [file 41598_2021_1583_MOESM1_ESM.docx]

| Kernel | Parameter | Distribution |
| --- | --- | --- |
| Compound exponential | K_e_ | uniform(0, 1) |
|  | a_1_ | uniform(0, 2) |
|  | a_2_ | uniform(0, a1) |
|  | D | uniform(1, 10) |
|  | k | uniform(0, 10) |
| Modified Cauchy | β | uniform(0.63, 5) |
|  | c_1_ | uniform(0, 1) |
|  | D | uniform(1, 10) |
|  | k | uniform(0, 10) |
| ZIP observation model |  |  |
|  | b_1_ | normal(0, 1000) |
|  | b_2_ | normal(0, 1000) |

**Supplementary Table S1.** Prior distribution of parameters for kernels and zero-inflated Poisson (ZIP) observation model.

| Distance (m) | 2009-1 | | 2009-2A | | 2009-2B | | 2010-1 | |
| --- | --- | --- | --- | --- | --- | --- | --- | --- |
|  | Obs (%) | Prob (%) | Obs (%) | Prob (%) | Obs (%) | Prob (%) | Obs (%) | Prob (%) |
| 0.75 | 0.0 | 0.0 | 0.0 | 0.0 |  |  |  |  |
| 1.5 | 0.0 | 0.0 | 0.0 | 0.0 |  |  |  |  |
| 2.25 | 0.0 | 0.0 | 0.0 | 0.0 |  |  |  |  |
| 3 | 0.0 | 0.0 | 0.0 | 0.0 |  |  |  |  |
| 3.75 | 0.0 | 0.0 | 0.0 | 0.0 |  |  |  |  |
| 4.5 | 0.0 | 0.0 | 0.0 | 0.0 |  |  |  |  |
| 5.25 | 0.0 | 0.0 | 0.0 | 0.0 |  |  |  |  |
| 6 | 0.0 | 0.0 | 0.0 | 0.0 |  |  |  |  |
| 6.75 | 0.0 | 0.0 | 0.0 | 0.0 | 0.0 | 0.0 |  |  |
| 7.5 | 0.0 | 0.0 | 0.0 | 0.0 | 0.0 | 0.0 | 0.0 | 0.0 |
| 8.25 | 5.0 | 0.1 | 0.0 | 0.0 | 0.0 | 0.0 | 0.0 | 0.0 |
| 9 | 0.0 | 0.0 | 0.0 | 0.0 | 0.0 | 0.0 | 0.0 | 0.0 |
| 9.75 | 5.0 | 0.0 | 0.0 | 0.0 | 0.0 | 0.0 | 3.0 | 0.0 |
| 10.5 | 0.0 | 0.0 | 0.0 | 0.0 | 0.0 | 0.0 | 0.0 | 0.0 |
| 11.25 | 0.0 | 0.0 | 0.0 | 0.0 | 0.0 | 0.0 | 0.0 | 0.0 |
| 12 | 0.0 | 0.0 | 0.0 | 0.0 | 0.0 | 0.0 | 5.6 | 0.1 |
| 18 | 20.0 | 6.1 | 0.0 | 0.0 | 0.0 | 0.2 | 10.5 | 6.1 |
| 24 | 0.0 | 1.0 | 0.0 | 0.1 | 5.0 | 0.5 | 19.4 | 22.3 |
| 30 | 20.0 | 12.9 | 0.0 | 0.1 | 0.0 | 0.6 | 31.6 | 26.8 |
| 36 | 15.0 | 18.3 | 0.0 | 1.0 | 0.0 | 5.5 | 45.9 | 41.0 |
| 42 | 35.0 | 42.7 | 5.0 | 9.5 | 5.0 | 7.1 | 57.1 | 54.9 |
| 48 | 25.0 | 33.3 | 15.0 | 6.4 | 5.0 | 12.9 | 55.3 | 50.4 |
| 54 | 35.0 | 52.2 | 15.0 | 11.1 | 20.0 | 28.7 | 65.7 | 34.7 |
| 60 | 60.0 | 67.0 | 21.1 | 11.6 | 28.6 | 38.6 | 76.3 | 54.6 |
| Percent of zero-excess condition (%) | 16.7 | | 12.5 | | 6.25 | | 60 | |

**Supplementary Table S2.** The observed (Obs) zero cross-pollination (CP) grain event percentage and the probability (Prob) of zero CP grain event under the Poisson distribution at different distances in all experiments.

| Model code | DIC |
| --- | --- |
| ZExpoN | 45856 ± 639 |
| ZExpoB | 35766 ± 791 |
| ZCauchyN | 43327 ± 824 |
| ZCauchyB | 36576 ± 1094 |

**Supplementary Table S3.** The average mean and standard deviation of deviance information criterion (DIC) of dispersal models obtained using 3-fold cross-validation.
